# Composite Electrolyte Membranes from Partially Fluorinated Polymer and Hyperbranched, Sulfonated Polysulfone

**DOI:** 10.3390/nano4010001

**Published:** 2013-12-23

**Authors:** Surya Subianto, Namita Roy Choudhury, Naba Dutta

**Affiliations:** Ian Wark Research Institute, University of South Australia, Mawson Lakes Campus, 5095 Adelaide, Australia; E-Mails: surya.subianto@univ-montp2.fr (S.S.); naba.dutta@unisa.edu.au (N.D.)

**Keywords:** membrane, fluoropolymer, polymer electrolyte, hyperbranched polysulfone, polymer blends

## Abstract

Macromolecular modification of poly(vinylidene fluoride-co-hexafluoropropylene) (PVDF) was done with various proportions of sulfonic acid terminated, hyperbranched polysulfone (HPSU) with a view to prepare ion conducting membranes. The PVDF-co-HFP was first chemically modified by dehydrofluorination and chlorosulfonation in order to make the membrane more hydrophilic as well as to introduce unsaturation, which would allow crosslinking of the PVDF-co-HFP matrix to improve the stability of the membrane. The modified samples were characterized for ion exchange capacity, morphology, and performance. The HPSU modified S-PVDF membrane shows good stability and ionic conductivity of 5.1 mS cm^−1^ at 80 °C and 100% RH for blends containing 20% HPSU, which is higher than the literature values for equivalent blend membranes using Nafion. SEM analysis of the blend membranes containing 15% or more HPSU shows the presence of spherical domains with a size range of 300–800 nm within the membranes, which are believed to be the HPSU-rich area.

## 1. Introduction

Macromolecular modification of polymer offers an efficient route to modify wettability, morphology, and performance of the resultant blend or composite systems due to its simplicity of operation and cost. Modifying a non-conducting polymer through conductive inclusion or using a conducting polymer can lead to enhanced hydrophilicity and conductivity, and, hence, can tailor resultant material performance. Careful selection of the component polymer or conducting inclusion can improve the excessive swelling of the conducting component, on the other hand enhance the conductivity of the non-conducting component in a beneficial way.

Polyvinylidene fluoride co-hexafluoropropylene, PVDF-co-HFP, is a partially fluorinated copolymer that has good chemical, mechanical, and thermal stability [1]. Due to its low cost there have been many studies into its potential use as a solid electrolyte or proton exchange membrane, however the material itself is insulating and ionic functionalities have to be added to the polymer to impart proton conductivity. Some studies have used chemical grafting [2,3,4,5,6] to attach acidic moieties to PVDF-co-HFP, while others have synthesized partially fluorinated block copolymers containing aromatic units that can be sulfonated [7]. Another, potentially more cost-effective approach is to blend the PVDF-co-HFP with various hydrophilic, proton conducting materials [8,9,10,11,12,13,14,15], such as Nafion™ or hydrophilic inorganic particles. Blends of miscible polymers often produce synergistic effects [9,16], and they can often be rapidly prepared through simple casting methods.

In order to achieve a blend membrane with good conductivity, the hydrophilic component of the blend needs to have a very high degree of functionality. Indeed, in a previous study of PVDF-co-HFP/Nafion blends [12], it was found that PVDF blends with Nafion of lower equivalent weight show greater conductivity due to the increased functionality. Hyperbranched polymers have attracted a great deal of attention in various fields due to their unique properties. Their dendritic structure and large number of end groups provide very high functionalities, and they can be synthesized in one step from AB*_x_* type monomer (*x* = 2 or more) [17,18,19,20]. Many previous studies [18,19,21,22,23,24,25] have synthesized hyperbranched, sulfonated polysulfones, including some studies regarding their potential use in proton-conducting membranes. However, they often have poor mechanical properties and as such unsuitable for use in membranes that require a robust material. To overcome this, the hyperbranched polymers have generally been either grafted or blended with another polymer that would serve as a structural support.

As PVDF-co-HFP is hydrophobic, direct blending with highly hydrophilic polymers may result in excessive phase separation and poor morphology, as previous studies with PVDF blends often have shown to require some chemical modification [9] or compatibilizer [8,10]. In order to achieve a good blend with a highly hydrophilic, hyperbranched polysulfone, the PVDF-co-HFP polymer needs to possess some hydrophilicity. Previous studies have shown that PVDF-co-HFP can be modified by dehydrofluorination [4,9,26,27] with alkali, which results in conjugated double bonds in the vinylidene fluoride chain. Other studies have also shown that PVDF-co-HFP can be made more hydrophilic by sulfonation, either through dehydrofluorination [27] or directly with chlorosulfonic acid [28].

In this paper, we present a study of an ion-conducting composite membrane containing sulfonic acid terminated hyperbranched polysulfone in a partially sulfonated and dehydrofluorinated PVDF-co-HFP matrix. The composite membranes show good compatibility between the PVDF-co-HFP matrix and the hyperbranched polysulfone, as well as good thermal stability and ionic conductivity.

## 2. Experimental

### 2.1. Materials

All chemicals were purchased from Aldrich (Castle Hill, Australia). Dimethyl sulfoxide (DMSO) was distilled under reduced pressure over calcium hydride before use.

### 2.2. Dehydofluorination of PVDF-co-HFP

PVDF-co-HFP (average molecular weight 400,000, 10 g) was dissolved in dimethyl acetamide (DMAc) (150 mL), and 10 mL solution of 0.05 M NaOH in isopropanol (IPA) was slowly added dropwise with vigorous stirring at room temperature. The solution was then stirred for a further 15 min and then precipitated in water. The polymer was filtered and rinsed several times with deionized water and dried under vacuum.

### 2.3. Synthesis of Chlorosulfonated PVDF-co-HFP(S-PVDF)

Dehydrofluorinated PVDF-co-HFP (5 g) was dissolved in 1-methyl-2-pyrrolidinone (150 mL) and chlorosulfonic acid (15 mL) was added dropwise. The solution was kept in an ice bath during addition of chlorosulfonic acid and then stirred overnight at room temperature. The sulfonated PVDF-co-HFP (S-PVDF) was then precipitated in water, filtered, and rinsed thoroughly with water and dried under vacuum.

### 2.4. Synthesis of Sulfonic Acid Terminated, Hyperbranched Polysulfone (HPSU)

Sulfonic acid terminated, hyperbranched polysulfone (HPSU) was synthesized according to the method described by Takeuchi *et al.* [19]. In a typical synthesis, 1 g of 2,6-bis(p-sodiumphenoxy) benzonitrile [19] was suspended in 5 mL PPMA (phosphorus pentoxide/methanesulfonic acid) and the solution was put under vacuum for 30 min. The solution was then purged with nitrogen and stirred at 150 °C for 24 h, after which it was poured onto deionized water to quench the reaction. The solution was then dialyzed for 3 days (see supporting information for molecular weight) and dried under vacuum, resulting in dark brown solid. ^1^H NMR (DMSO-d_6_): broad multiplets at 6.4–8.1 ppm.

### Ion Exchange Capacity

Ion exchange capacity (IEC) was obtained by soaking the samples in 0.02 M NaOH overnight and titrating the solution with standardized 0.02 M HCl using phenolphthalein indicator. The IEC was obtained based on the difference in the volume of HCl required to neutralize the solution compared to a blank with no sample present (*V*_blank_ − *V*_sample_), and was calculated according to Equation (1).


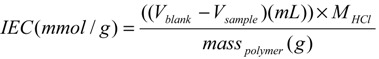
(1)

Matrix assisted laser desorption ionization (MALDI) time of flight mass spectroscopy (ToF-MS) analysis. Samples were analyzed using MALDI-ToF-MS (Micromass M@LDI LR Instrument from Waters, Manchester, UK) equipped with a pulsed (4 ns) nitrogen laser emitting at 337 nm. The detector was operated in positive ion mode. The pulse voltage was set to 1523 V. At least 20 spectra were collected and combined.

### 2.5. Casting of the S-PVDF/HPSU Composite

HPSU was dissolved in DMSO/H_2_O (19.5:0.5 *v*/*v*, 20 mL) and added to a solution of S-PVDF (1 g) in DMAc (100 mL). The solution was stirred at room temperature for 15 min and cast onto Teflon moulds and dried at 120 °C overnight. The films were then annealed at 80 °C for another 24 h. The films were then soaked in 0.1 M H_2_SO_4_ for 24 h to ensure acidification of the HPSU, and finally rinsed with deionized water to remove excess acid.

### Water Uptake Measurements

Weighed dry membranes were immersed in distilled water for 24 h. The membranes were removed from water, gently blotted between tissue paper to remove surface water, and weighed. This water uptake measurement was repeated in three different samples and the average values are reported. The water uptake (*U*) was calculated using the weights of wet membrane (*W_w_*) and dry membrane (*W_d_*) using the following Equation:

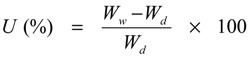
(2)

### 2.6. Spectroscopic Analysis

Photo-Acoustic Fourier Transform Infrared Spectroscopy (PA-FTIR, MTEC Photoacoustics Inc, Ames, IA, USA) was performed on a Nicolet Magna-IR Spectrometer 750 (Thermo Fisher Scientific Inc, Waltham, MA, USA) equipped with an MTEC model 300 photoacoustic cell under helium purge with carbon black as the reference. Nuclear Magnetic Resonance Spectroscopy (NMR, Bruker Corporation, Fällanden, Switzerland) was performed using a Bruker 600 MHz NMR with Bruker TOPSPIN software (Bruker Corporation, Fällanden, Switzerland). XPS (X-ray photoelectron spectroscopy) was done on a Kratos Axis Ultra (Kratos Analytical Ltd., Manchester, UK) with an Al source and the data was analyzed using the CasaXPS program. Elemental analysis using XPS was performed by peak area analysis of a survey scan of the samples.

### 2.7. Thermal Analysis

Thermogravimetric Analysis (TGA) was done on a TA 2950 Thermal Analyzer (TA Instruments, New Castle, PA, USA). Samples were dried in an oven at 80 °C prior to analysis and the experiment was performed on approximately 5 mg of the sample under nitrogen with a temperature ramp of 10 °C min^−^^1^. DSC was performed using a TA 2920 DSC instrument (TA Instruments, New Castle, PA, USA) with a heating rate of 10 °C min^−1^ under a nitrogen atmosphere. The sample mass was kept between 5 and 10 mg. The unit was fitted with a liquid nitrogen cooling accessory (LNCA). Dry samples were cooled from room temperature down to −50 °C, kept at the temperature isothermally for 5 min, and then heated up to 300 °C.

Dynamic mechanical properties of the samples were measured using DMA 800 (TA Instruments, New Castle, PA, USA) in tension mode with a typical dimension of 15 mm × 6 mm × 0.2 mm. Samples were heated from room temperature to 75 °C under humidity at a frequency of 1 Hz, at 0.08% strain amplitude with a programmed heating rate of 3 °C min^−1^.

### 2.8. Conductivity Measurement

In-plane proton conductivity measurements were performed by AC impedance using a Solartron 1260A Electrochemical Analyzer (Solartron Metrology Ltd., Leicester, UK) in a 2-electrode setup. The films were cut into a 5 mm × 20 mm × 0.5 mm strip and the measurements were performed in an ESPEC 2930 controlled humidity chamber. The impedance of the film was then calculated as per Equation (3):

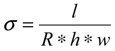
(3)
where *l* is the distance (cm) between the two Pt electrodes, *h* and *w* are the thickness (cm) and width (cm) of the membrane respectively, and *R* (Ω) is the resistance of the membrane obtained from the complex impedance plot. The measurements were done in duplicate with an error margin of 5%.

### 2.9. Elemental Analysis

Elemental analyses of S-PVDF were performed at University of Queensland’s Microanalytical services (Brisbane, Australia).

### 2.10. Scanning Electron Microscopy (SEM) Energy Dispersive X-Ray (SEM-EDAX)

A Philips XL30 SEM (FEI Company, Oregon, OR, USA) coupled with energy dispersive X-ray analyser were used to obtain all SEM images of the blends. Both the surface and cross section was imaged for the samples and all samples were carbon coated before imaging. The microscope was operated at an acceleration voltage of 20 kV and all images were scanned using the slow scan mode for improved clarity.

## 3. Results and Discussion

### 3.1. Synthesis of HPSU

**Scheme 1:**
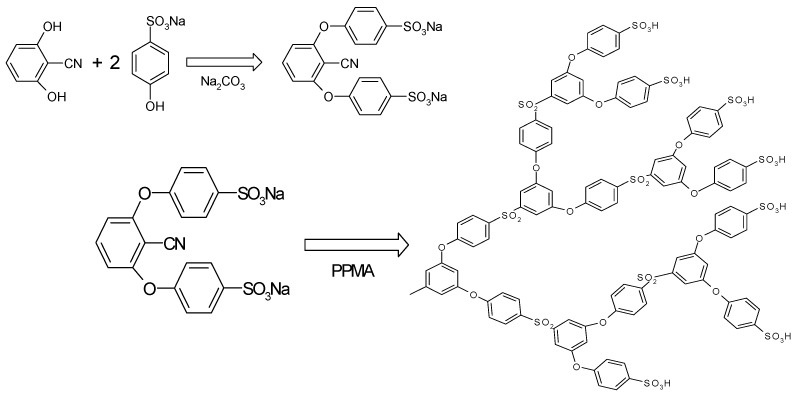
Synthesis of the sulfonic acid terminated, hyperbranched polysulfone.

Sulfonic acid-terminated, hyperbranched polysulfone (HPSU) was synthesized based on the procedure in Scheme 1 described by Takeuchi *et al.* [18,19]. However, the authors did not report any detail of the effect of various synthetic parameters, and, in our study, we examined the effect of time and temperature and found that the reaction temperature has significant influence in determining the yield and nature of product obtained.

**Figure 1:**
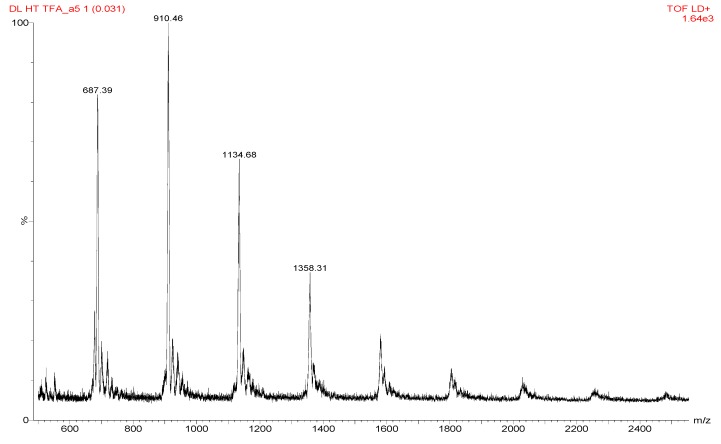
MALDI spectrum of the synthesized HPSU.

Figure 1 shows the MALDI spectrum on synthesized and dialysed samples of HPSU, which shows molecular weight values. In general, the characterization of branched polymers is challenging, as reported by earlier authors [20], because (even in the hypothetical case of a size exclusion chromatography system without band broadening) a sample of branched polymers with the same *V*_h_, hydrodynamic volume, in general, contains chains with a range of molecular weights. This effect is called variously “imperfect resolution”, “structural polydispersity”, or “local polydispersity”. Matrix assisted laser desorption ionization spectroscopy, MALDI was performed using dialyzed (dialysis bag cut off molecular weight 12,000) samples, however, the molecular weight obtained was very low, around <2000, showing only the lower molecular weight oligomers. The MALDI spectrum shows a repeating unit of a mass around 224–226, which is a bit higher (or lower) than expected from theoretical fragment (217 or 233 based on cleavage on the ether and assuming loss of CN group). The molecular weights of the synthesized samples have a broad distribution from 1000 to 12,000.

The HPSU was synthesized with high yields when the reaction was carried out at 150 °C and above, however with increasing temperature there is a decrease in the IEC and water solubility of the HPSU. As can be seen in Table 1, the IEC of the product decreases when the reaction temperature or time is increased. This is likely due to an annealing effect, with higher temperatures resulting in more aggregation and crosslinking between the polymer chains, resulting in smaller number of sulfonic acid end groups (lower IEC) and lower solubility. Despite the lower water solubility, samples synthesized at higher temperature were still soluble in DMSO/H_2_O mixture.

**Table 1:** Effect of reaction temperature and time on the ion exchange capacity (IEC) of the hyperbranched polysulfone (HPSU).

Temperature (°C)	Time (days)	IEC (meq/g)
130	3	5.0
140	1	5.2
140	2	4.9
150	1	4.2
150	2	3.9

The use of higher temperature also increased the yield, with the yield increasing significantly from 20% (at 130 °C) to 80%–90% (at 150 °C) as purification was done through dialysis, and thus the increased annealing and crosslinking would result in larger aggregates, which are retained within the dialysis tube. In this study, it has been determined that a synthesis parameter of 150 °C and 24 h provides the optimum product in terms of yield, solubility, and IEC. As such, this condition has been used to synthesize the HPSU used in the composite membrane.

**Figure 2:**
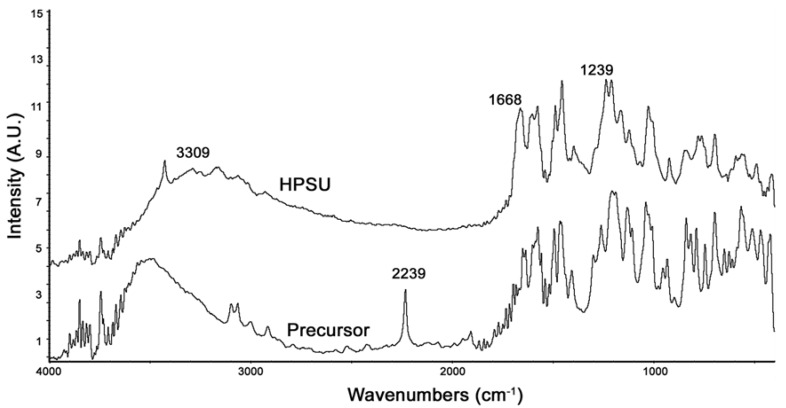
PA-FTIR spectra of the HPSU and its precursor compounds.

FTIR of the synthesized HPSU is shown in Figure 2. It shows that the large, broad peak at 3300–3500 cm^−^^1^ due to OH groups has been shifted in the HPSU compared to the precursor, and there are also shifts in the peaks at 1668 and 1239 cm^−^^1^ due to the sulfone linkages. The lack of a peak at 2239 cm^−^^1^ indicates that the nitrile group present in the precursor has been lost during polymerization.

**Figure 3:**
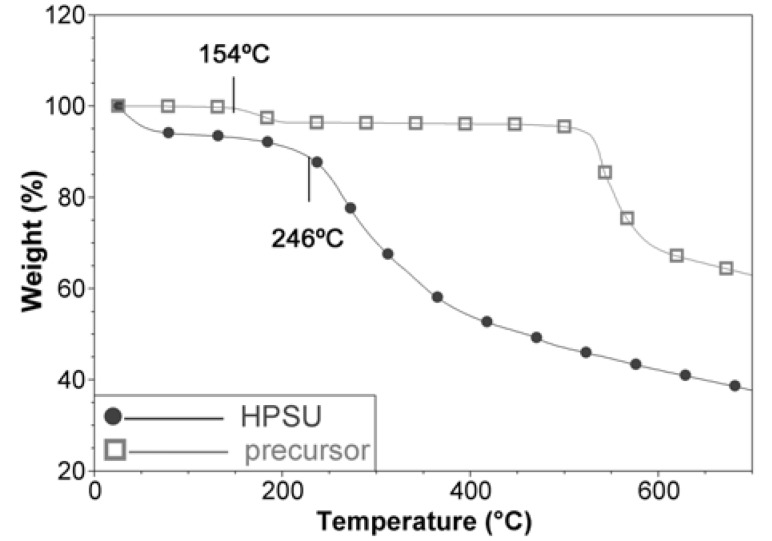
TGA comparison of HPSU and its precursor compound.

This is consistent with the TGA analysis in Figure 3, which shows a mass loss at 154 °C, assigned to the nitrile group for the precursor, but this mass loss is absent in the HPSU. The HPSU shows that it is thermally stable until 246 °C, where desulfonation occurs (This is not observed in the precursor as it is in sodium salt form), but there were no well-defined mass loss for main chain degradation, with the curve sloping downwards as temperature increased. This indicates that the HPSU is not of a well-defined structure, and most likely is a mixture of hyperbranched polymers of varying branch length. This non-uniformity is unlikely to affect the performance of the blend membrane, as the HPSU’s purpose is to provide proton conductivity (which would rely on sulfonic acid groups) and the mechanical and structural integrity of the membrane would be provided by the PVDF-co-HFP matrix.

### 3.2. Sulfonation of PVDF-co-HFP

**Scheme 2:**
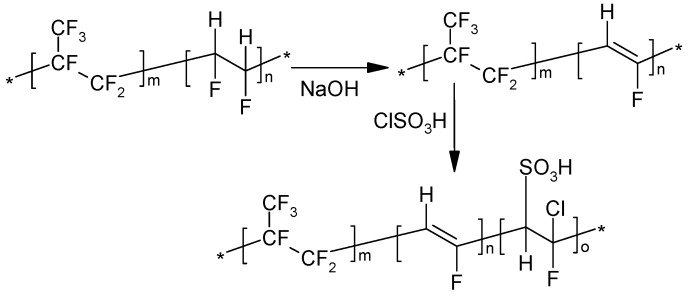
Sulfonation of PVDF-co-HFP.

PVDF-co-HFP was modified by sulfonation in order to introduce some hydrophilicity onto the material and improve its compatibility with the sulfonic acid-terminated HPSU. As shown in Scheme 2, the sulfonation was achieved through dehydrofluorination of the vinylidene fluoride moieties followed by the addition of chlorosulfonic acid to the double bonds. The dehydrofluorination was done in DMAc using dilute solutions in order to prevent formation of black, insoluble product as was observed by Bottino *et al.* [27] This precipitation is likely due to highly conjugated, crosslinked product, which traps the alkali solvent (normally an alcohol) in which the PVDF-co-HFP is insoluble. It was also found that the use of isopropyl alcohol instead of methanol and vigorous stirring, combined with slow addition of the reactant was necessary to prevent precipitation of the product.

Previous studies on the sulfonation of PVDF-co-HFP [27,28] used concentrated acids as they were performed on polymer solids, however, such procedure is likely to give rise to non-uniformity within the material, and, thus, in this study the sulfonation was done in solution. Chlorosulfonic acid was chosen due to its high reactivity, and the sulfonation was done in dilute solutions of the dehydrofluorinated PVDF-co-HFP in 1-methyl-pyrrolidinone (NMP) with vigorous stirring in order to prevent precipitation of the sulfonated product. High temperature was not used in order to prevent crosslinking and gelling of the dehydrofluorinated PVDF-co-HFP.

The sulfonated PVDF-co-HFP (S-PVDF) is soluble in DMAc and NMP and shows a small water uptake and IEC, but due to the low value of the IEC (<0.1 meq/g) it could not be accurately measured. It appears that due to the mild reaction conditions and low temperature used, only a small amount of sulfonation was achieved. This means that the majority of the double bonds remain unsulfonated (and unreacted as the S-PVDF remains highly soluble in DMAc), which in this case is desirable as they would be available for crosslinking during the casting of the composite membrane, which would help to immobilize the HPSU within the S-PVDF matrix.

**Figure 4:**
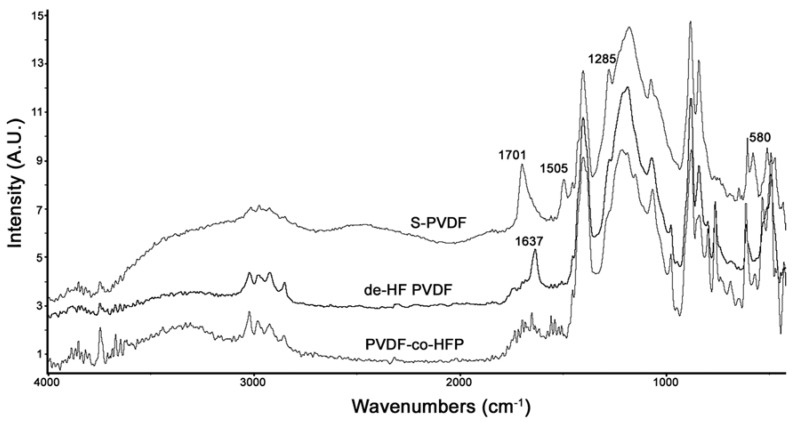
Photoacoustic FTIR spectra comparison between PVDF-co-HFP, dehydrofluorinated PVDF-co-HFP, and S-PVDF.

Figure 4 shows the comparison between the PA-FTIR spectra of PVDF-co-HFP, dehydrofluorinated PVDF-co-HFP, and S-PVDF. As can be seen, dehydrofluorination results in a new peak at 1637 cm^−^^1^ due to the double bonds. After sulfonation, this peak has broadened and shifted to 1701 cm^−^^1^. Sulfonation also resulted in a very broad peak at 3300–3500 cm^−^^1^ and a peak at 1285 cm^−^^1^ which is attributed to the sulfonic acid group. The peaks at 1505 and 580 cm^−^^1^ are likely due to the presence of C–Cl bands present due to the use of chlorosulfonic acid.

**Figure 5:**
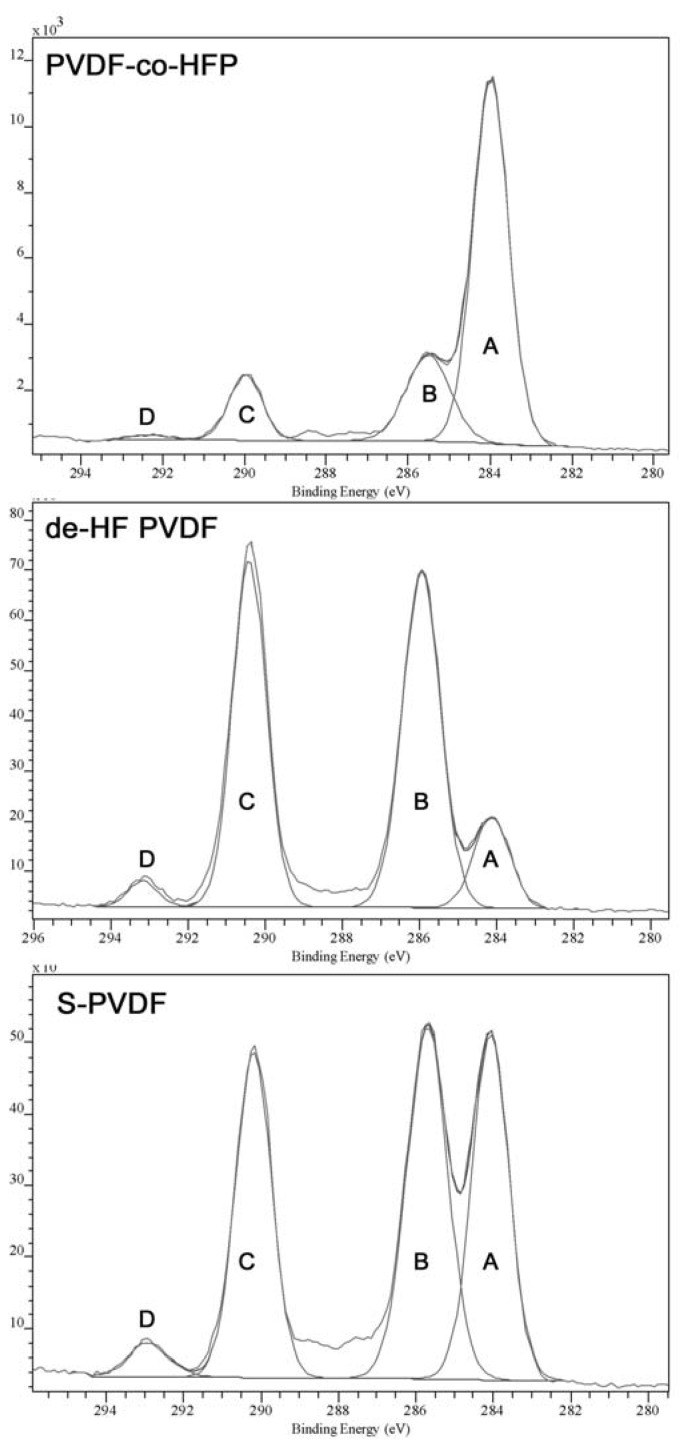
High resolution XPS C1s spectrum of PVDF-co-HFP, dehydrofluorinated PVDF-co-HFP, and S-PVDF.

The low IEC value is due to the very low degree of sulfonation, which has been quantified through XPS and elemental analysis. Both analyses methods show only a small amount of sulfur in the material. Elemental analysis shows a 0.1%–0.3% of sulfur in the material, while XPS shows 0.2%–0.4% of sulfur. The XPS high resolution C1s spectra comparison also shows significant difference between the PVDF-co-HFP samples after dehydrofluorination or sulfonation. The peak fitting of C1S is given in Figure 5, which shows that the spectrum has four main peaks at around 284, 285.5, 290, and 292.5 eV, labelled “A” to “D”, respectively. Although the actual number of peaks in the spectrum is likely to be higher, extra peaks may produce greater inaccuracies in the peak fitting and thus the analysis will be concerned mainly with the four main peaks, which are quite well-defined. As can be seen from Table 2, chemical modification of the sample has resulted in Peak D becoming more prominent as well as being shifted to higher B.E. (binding energy), which is attributed to the effect of double bonds or sulfonic acid groups present in the modified polymer.

**Table 2:** Binding energy assignments in high-resolution C1s XPS.

Peak Label (Approx. B.E.)	Functional Group Assignment	B.E. (PVDF-co-HFP, Aldrich)	B.E. (dehydro fluorinated PVDF-co-HFP)	B.E. (S-PVDF)
A (284 eV)	–C–C–	284 eV	284 eV	284 eV
B (285 eV)	–HCF–	285.5 eV	285.9 eV	285.7 eV
C (290 eV)	–CF_2_–, –HC=	290.0 eV	290.4 eV	290.2 eV
D (292 eV)	–CF_3_, –FC=, –FC–SO_3_H	292.3 eV	293.2 eV	292.9 eV

Table 3 shows the comparison of the peak area ratio between Peak B (predominantly influenced by vinylidene fluoride) and Peak C (predominantly influenced by hexafluoropropylene) also shows that dehydrofluorination is occurring with Peak B becoming less prominent as the vinylidene fluoride segments are dehydrofluorinated.

**Table 3:** Peak area ratio of Peaks B and C in the high-resolution C1s spectra.

Sample	Peak Area Ratio (Peak B/Peak C)
PVDF-co-HFP	1.72
De-HF PVDF	1.09
S-PVDF	1.22

### 3.3. Casting of SPVDF-HPSU Composite Membrane

The S-PVDF/HPSU blend membranes were made by solvent casting from a mixture of DMAc (for S-PVDF) and DMSO (for HPSU). The two solutions were fully miscible, and there were no sign of phase separation during casting. The solvents were chosen for their miscibility and similar boiling points, which would prevent one solvent being completely removed before the other. Casting of the composite was done at high temperature to drive off the solvent and promote annealing of the sample, as subjecting SPVDF to high temperature promotes cross-linking in the sample and reduces their solubility. Sample dried at 120 °C shows good stability, and after annealing it also shows only a very small mass loss (<1%) upon leaching in water. The composite membrane also shows increased water uptake compared to blank S-PVDF membranes. 

**Figure 6:**
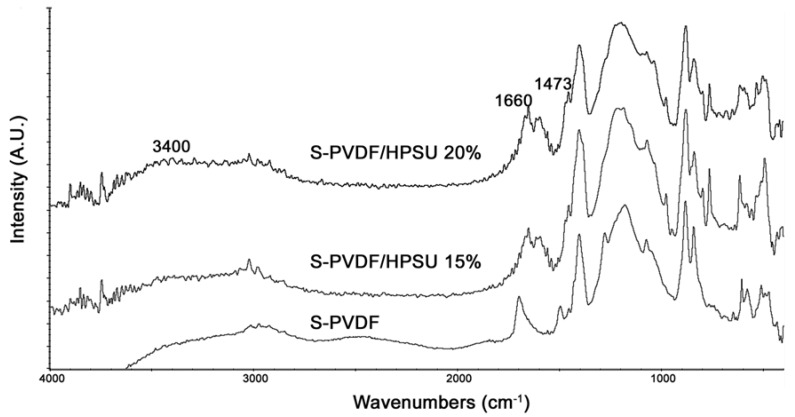
PA-FTIR Spectra of S-PVDF and the S-PVDF/HPSU composite.

FTIR spectrum of the composite in Figure 6 shows an increase in the peak at 3400, 1660, and 1473 cm^−^^1^ with increasing hyperbranched PSU content due to the sulfonic acid and sulfone functionalities in the HPSU. The peak due to unsaturation at 1700 cm^−^^1^ has also appeared to be suppressed, which indicates that annealing of the sample have promoted crosslinking of the PVDF-co-HFP matrix.

**Figure 7:**
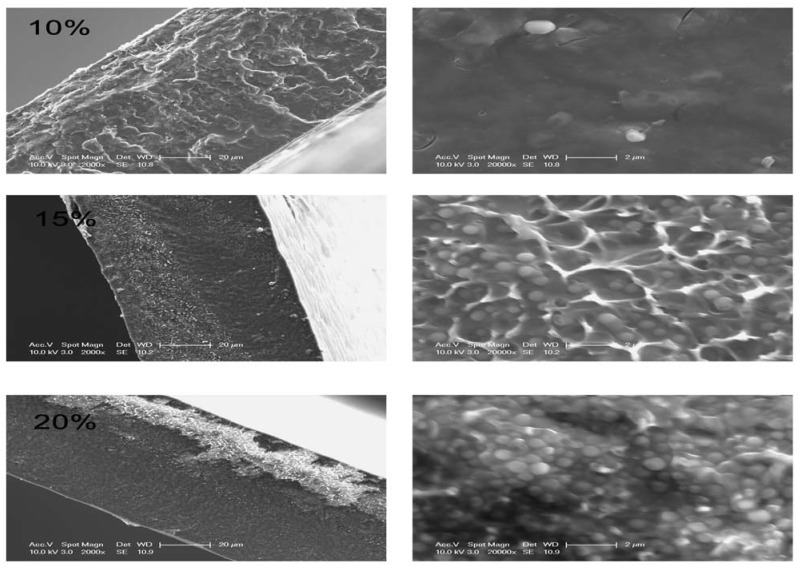
Cross section SEM images of the S-PVDF/HPSU composite membranes with 10%, 15% (volume %) HPSU (magnifications: left images 20 μm; right images: magnified view at 2 μm). Sample cross section was obtained by fracturing the membranes in liquid N_2_.

The composite was cast as a continuous film without any visual evidence of phase separation between the S-PVDF and HPSU in solution during casting. SEM images of the cross section of the film in Figure 6 (left column images at a magnification of 20 μm) show a smooth, continuous film, without pores or excessive phase separation between the S-PVDF and HPSU. However, as can be seen in Figure 7 (right column images at a magnification of 2 μm), there were some spherical domains, which were observed for samples containing 15% HPSU or more. The domains appear to be uniformly distributed and of similar size regardless of HPSU content. As can be seen from Figure 7, the spherical domains are typically around 300–800 nm in diameter. The number of these spherical domains visible increases with HPSU content, indicating that the observed morphology is due to the HPSU.

As the PVDF-co-HFP was sulfonated in the vinylidene fluoride regions, these domains would be due to phase separation between polar areas of predominantly vinylidene fluoride/HPSU and nonpolar, hexafluoropropylene areas. The number of the spherical domains seems to increase with HPSU content as the HPSU forms the spherical domain. EDX analysis in Table 4 shows an increase in sulphur content on the spherical domains compared to the matrix, confirming that the HPSU is contained within these domains. The presence of such spherical domains of quite uniform shape and size is of interest, as it has not been commonly observed with PVDF/ionomer blends, however, the spherical phase separation is similar to what has been observed with grafted [22] or block [7] polysulfone/PVDF composite. Such domains may have arisen due to the annealing process, as aggregates of HPSU are trapped by the crosslinking of the PVDF matrix. This is because the site for dehydrofluorination and sulfonation both relies on vinylidene fluoride segments, with hexafluoropropylene segments being inert and unchanged and, thus, acting as the matrix linking the domains.

**Table 4:** Elemental analysis of a composite membrane containing 15% HPSU by SEM EDX.

Area Analysed	Sulphur Content (Atom %)
Matrix	3.91
Spherical Particles	5.95

**Figure 8:**
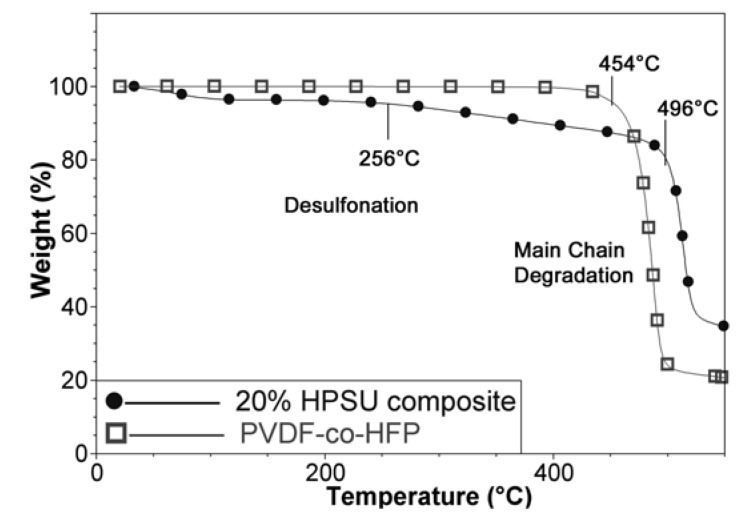
TGA spectra of the blend membrane (20% HPSU) and untreated PVDF-co-HFP.

TGA analysis of the composite membrane shows two decomposition temperatures around 260 °C and 490 °C due to desulfonation and main chain degradation respectively. The appearance of water loss and desulfonation shows that the HPSU has been incorporated in the material, as S-PVDF alone contains too little sulfonic acid groups to show appreciable mass loss due to desulfonation. Furthermore, TGA comparison in Figure 8 shows that the main chain degradation in the composite membrane occurs at higher temperature (496 °C) than the main chain degradation temperature of 450 °C for untreated PVDF-co-HFP. This is attributed to annealing and crosslinking of the residual double bonds present due to dehydrofluorination (as only a small amount are sulfonated), resulting in higher thermal stability of the composite membrane.

**Figure 9:**
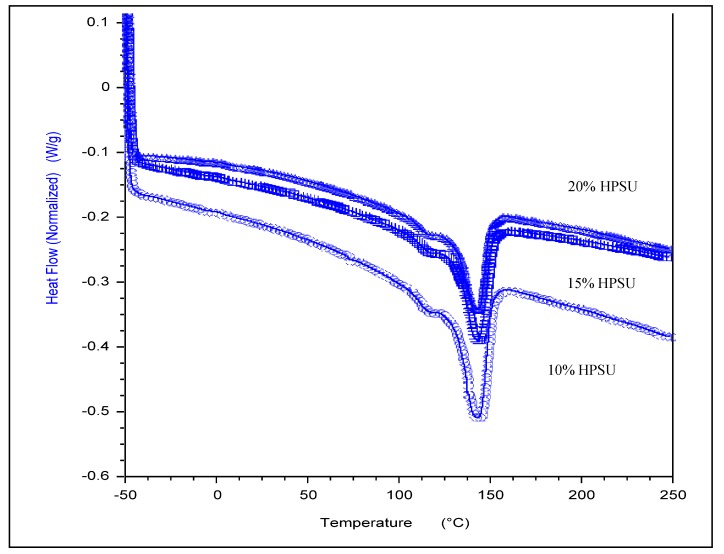
DSC thermograms of the blend membranes (10%, 15%, 20% HPSU) with S-PVDF.

DSC curves for blend membranes under nitrogen in the range of −50 to 250 °C are shown in Figure 9. Pure PVDF-HFP shows only a melting peak at 140 °C. The membranes show a large endothermic peak at 140 °C. This peak is related to the *T*_m_ of PVDF-HFP. The small endothermic peak ~110 °C in all the samples is due to release of bound water, as also evidenced by TGA results, which is associated with hydrophilic HPSU content, as it can retain greater content of water molecules (2%–5%).While the slight lowering of enthalpy of melting (*T*_m_) for the fluoropolymer may indicate a small degree of mixing of minor component of HPSU with the PVDF-HFP amorphous segments, the blends are essentially incompatible. Therefore, phase separation between HPSU and PVDF-HFP contributes to the formation of droplet structure of HPSU in the PVDF-HFP matrix.

**Figure 10:**
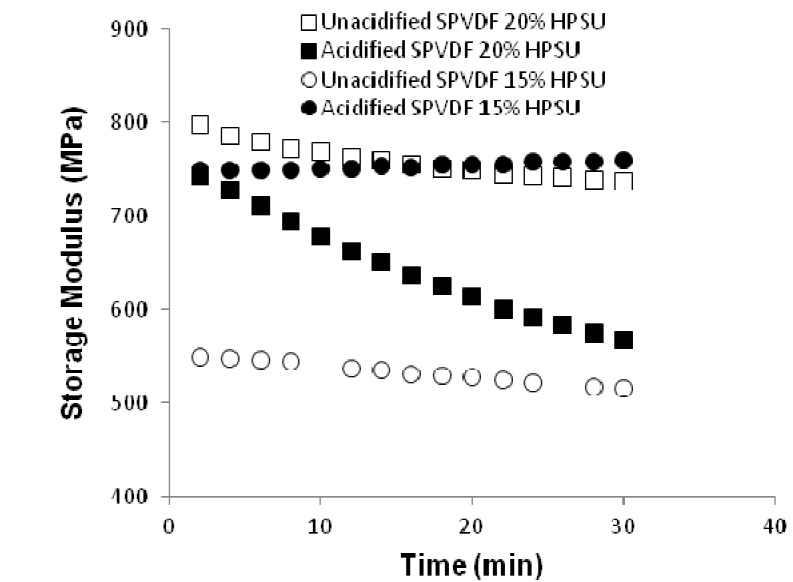
Storage moduli change of different blends at a constant humidity of 50%.

**Figure 11:**
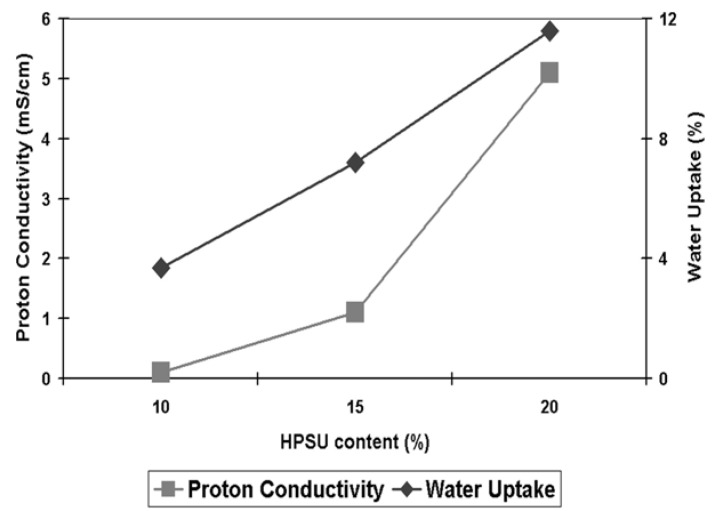
Ionic conductivity at 80°C and 100% humidity and water uptake of samples containing 10%–20% HPSU.

**Dynamic Mechanical Property:** Dynamic mechanical analysis was done using Q800 DMA under a constant humidity to investigate the effect of humidity on elastic modulus. Figure 10 shows the plot of elastic modulus *vs.* time at a relative humidity of 50%. It is clear that the unacidified blends with different contents of HPSU show marginal change in modulus with time, The modulus values of both the unacidified and acidified systems lie in the range of 800–500 MPa. However, an interesting feature has been observed for blend with 20% HPSU, which shows change in modulus over time under humidity. Such behavior can be accounted for the water absorption of this blend (~6% from Figure 11) than the other systems.

**Proton Conductivity:** As can be seen from Figure 11, the S-PVDF/HPSU membrane shows an increase in both proton conductivity and water uptake, with both values increasing with higher HPSU content. This is attributed to greater amount of acidic functionalities of the membrane resulting in greater hydrophilicity of the membrane. Samples with less than 10% HPSU were insulating, indicating that 10% HPSU content is the threshold required to achieve a continuous, conducting path throughout the membrane. However, unlike the water uptake which increases linearly, there was a greater increase in proton conductivity between the sample containing 15% and 20% HPSU. As both water uptake and ionic conductivity rely on the HPSU, the increase in HPSU would result in greater hydrophilicity and more acidic functionalities, allowing the membrane to uptake more water and provides greater conductivity. The water-retention capability of the blends can be improved by annealing at high temperature (120 °C) as it promotes aggregation and self-condensation within the ionic groups, which also lowers the solubility of the material. However, proton conductivity also relies on achieving an inter-connected pathway through the membrane, and as such the non-linear increase in conductivity is due to the HPSU content reaching the percolation threshold in the membrane, resulting in greater increase in the number of pathways for proton conduction. Indeed, it is likely that the spherical domains visible in the cross-section SEM images are HPSU rich-area and those domains were more visible in samples with higher HPSU content which also have higher proton conductivity. The highest conductivity value was obtained with the membrane containing 20% HPSU, which shows a conductivity of 5.1 mS cm^−^^1^ at 80 °C and 100% relative humidity.

Although the conductivity of the blend membrane is still much lower than that of Nafion™ (94 mS cm^−^^1^ for Nafion 117 tested under the same conditions), it is still quite significant and compares favorably to previous studies of PVDF blends. In particular, Cho *et al.* [2] reported that their Nafion™/PVDF-co-HFP achieved a value of 1.5 mS cm^−^^1^ for a 20% Nafion/PVDF-co-HFP blend at 80 °C and 100% RH, while Song *et al.* [9] reported that around 60%–70% Nafion content was required in their Nafion 115/PVDF blend to achieve similar conductivity to our membrane containing just 20% HPSU. This indicates that the greater density of functional group in the HPSU contributed to a better conductivity in the S-PVDF/HPSU membrane compared to blends using linear polymers, such as Nafion™.

The S-PVDF/HPSU blend also shows good morphology with no macroporosity and lower water uptake compared to the Nafion™/PVDF blend [12], which shows some pores at 20% Nafion content. The membrane also shows good stability with no loss of conductivity observed after soaking in water for two weeks. As this stability was observed mostly in annealed membranes, this is attributed to crosslinking of the PVDF matrix due to unsaturated sites resulting from dehydrofluorination trapping the HPSU within the membrane.

## 4. Conclusions

The present work demonstrates the potential of hyperbranched polymers for use as the conducting phase in a blend membrane. Their high number of end groups allows for greater degree of functionality compared to linear polymers, resulting in better ionic conductivity of up to 5.1 mS cm^−^^1^ than the literature values for equivalent Nafion™/PVDF blends. The HPSU was able to be immobilized in the PVDF-co-HFP matrix through dehydrofluorination and sulfonation of the matrix polymer which allows for some hydrophilicity as well as crosslinking of the membrane, enabling it to form a smooth, continuous blend with the HPSU, resulting in a blend with good stability. It was found that more than 15% HPSU was required to reach the percolation threshold in the membrane and achieve good ionic conductivity, and this is supported by cross sectional SEM of the membranes, which shows the presence of spherical domains in samples containing 15% and 20% HPSU. The spherical domains were around 300-800 nm in size, and were distributed quite evenly across the membrane.
